# Evolution in the Treatment of Psychiatric Disorders: From Psychosurgery to Psychopharmacology to Neuromodulation

**DOI:** 10.3389/fnins.2019.00108

**Published:** 2019-02-15

**Authors:** Michael D. Staudt, Eric Z. Herring, Keming Gao, Jonathan P. Miller, Jennifer A. Sweet

**Affiliations:** ^1^Department of Neurosurgery, University Hospitals Cleveland Medical Center, Case Western Reserve University, Cleveland, OH, United States; ^2^Department of Clinical Neurological Sciences, London Health Sciences Centre, Western University, London, ON, Canada; ^3^Department of Psychiatry, University Hospitals Cleveland Medical Center, Case Western Reserve University, Cleveland, OH, United States

**Keywords:** psychosurgery, lobotomy, psychiatric disease, depression, obsessive-compulsive disorder, Tourette syndrome, brain circuitry, deep brain stimulation

## Abstract

The treatment of psychiatric patients presents significant challenges to the clinical community, and a multidisciplinary approach to diagnosis and management is essential to facilitate optimal care. In particular, the neurosurgical treatment of psychiatric disorders, or “psychosurgery,” has held fascination throughout human history as a potential method of influencing behavior and consciousness. Early evidence of such procedures can be traced to prehistory, and interest flourished in the nineteenth and early twentieth century with greater insight into cerebral functional and anatomic localization. However, any discussion of psychosurgery invariably invokes controversy, as the widespread and indiscriminate use of the transorbital lobotomy in the mid-twentieth century resulted in profound ethical ramifications that persist to this day. The concurrent development of effective psychopharmacological treatments virtually eliminated the need and desire for psychosurgical procedures, and accordingly the research and practice of psychosurgery was dormant, but not forgotten. There has been a recent resurgence of interest for non-ablative therapies, due in part to modern advances in functional and structural neuroimaging and neuromodulation technology. In particular, deep brain stimulation is a promising treatment paradigm with the potential to modulate abnormal pathways and networks implicated in psychiatric disease states. Although there is enthusiasm regarding these recent advancements, it is important to reflect on the scientific, social, and ethical considerations of this controversial field.

## Introduction

The management of psychiatric disorders is challenging and often requires a multimodal approach to diagnosis and treatment. There is a rich history of innovation in the field, driven by scientists, physicians and surgeons. In particular, the neurosurgical treatment of psychiatric disorders has a long and tumultuous history fraught with controversy. However, the legacy of “psychosurgery” has also given rise to the development of modern standards for research and ethics, and has fostered a deeper understanding of the pathophysiology of human behavior. Although there is evidence of psychosurgery spanning multiple thousands of years throughout human history, the most exciting, but also provocative developments have been within the past century, attributable to the combined efforts of scientists and physicians. In the 1950s, ablative surgery fell out of favor due to the rise of effective pharmacology and intense professional and public criticism, although research and practice continued with more rigorous standards. Although the modern treatment of psychiatric disorders is primarily medical, the high incidence of treatment resistance and failure has fostered a renewed interest in surgical treatments with a non-ablative focus.

The aim of this article is to provide a brief chronological overview of the treatment of psychiatric diseases from ablation to pharmacology to neuromodulation. Despite a history of controversy, interest in the potential of surgery for psychiatric disorders has endured and even increased within the past few decades, primarily driven by the proliferation and success of neuromodulation and by improvements in structural and functional neuroimaging. It is important to view developments in psychosurgery in the context of the historical and current understanding of the neurobiology and pathophysiology of consciousness and behavior, the available treatments for psychiatric disorders, and the adherence to (or lack thereof) research ethics.

## A Historical Perspective

### The Origins of Psychosurgery

The earliest evidence of presumed psychosurgery has its origins in the Neolithic era; several skulls from this period have been identified with areas of trephination and evidence of healing (Stone and Miles, 1990; Alt et al., 1997), suggesting that these early procedures were likely performed with therapeutic intent. Although concurrent fractures have been identified in some specimens, numerous other skulls bear no obvious signs of trauma (Stone and Miles, 1990). It has been hypothesized that early trephination was performed for ritualistic or spiritual purposes, with the intent to treat manifestations of headaches, epilepsy, and mental illness (Robison et al., 2013). During the classical era, detailed guidelines regarding trephination, primarily for trauma, were outlined in the Hippocratic text “peri ton en cephali traumaton,” translated to “On Head Wounds” or “On Injuries of the Head” (Dimopoulos et al., 2008). Psychosurgery was also depicted in the medical literature and artwork of the Renaissance era, notably in “The cure of folly or the operation for the stone,” a painting by Hieronymus Bosch referring to the belief that madness was caused by a physical stone within the brain (Salcman, 2006).

The practice of psychosurgery was then largely absent from Western medicine for a few hundred years until its resurgence in the modern era. Throughout the 1800s, new insights into functional neuroanatomy and neurophysiology laid the foundation for renewed interest. In 1819, Franz Joseph Gall published his treatise on phrenology, which suggested that the brain possessed discrete functional regions (Simpson, 2005). Although phrenology was flawed and ultimately discredited, the idea of neurological functions having an anatomical correlate was expanded on by seminal work on the localization of language from Paul Broca and Carl Wernicke, and further work by Gustav Fritsch, Eduard Hitzig, and David Ferrier on localization of the motor cortex (Robison et al., 2013). The study of patients with traumatic brain injuries was also essential, the most famous example being Phineas Gage, who sustained a penetrating injury to his dominant frontal lobe and subsequently developed aggressive and impulsive behavior (Damasio et al., 1994).

### The Advent of Modern Psychosurgery

Inspired by these findings, Swiss psychiatrist Gottlieb Burckhardt performed the first psychosurgical treatments in 1888. These procedures, conducted on six patients with mental illnesses, consisted of topectomies to excise brain regions, thought to be responsible for aggression, agitation or hallucinations (Stone, 2001); Burckhardt was subsequently ostracized from the medical community. The research and practice of psychosurgery again entered into dormancy and was largely quiescent until 1935, when John Fulton and Carlyle Jacobsen described the role of the frontal lobe in short term memory, anxiety and aggression in a primate model (Fulton and Jacobson, 1935). In attendance of this lecture was Egas Moniz, a Portuguese neurologist who, together with the neurosurgeon Almeida Lima, developed the prefrontal leucotomy procedure for the treatment of psychiatric patients with prominent depression, anxiety or aggression (Feldman and Goodrich, 2001). This procedure initially consisted of alcohol injections into the prefrontal white matter, and Moniz eventually developed the leucotome, an instrument that could be introduced through frontal burr holes for precision lesioning (Moniz, 1994).

### The Prefrontal Lobotomy and Enduring Controversy

This work of Moniz and Lima inspired the Americans Walther Freeman, a neurologist, and James Watts, a neurosurgeon, who further refined the prefrontal leucotomy technique to allow for larger lesions that presumably disrupted widespread brain networks associated with affective processing. The precision leucotome was subsequently developed, and procedural modifications were introduced to allow for more accurate lesion localization using anatomical landmarks (Heller et al., 2008). This refined procedure was named the prefrontal lobotomy: minimal lobotomies were predominantly performed for the treatment of affective symptomatology, whereas radical lobotomies were for schizophrenic patients or those with refractory symptoms (Feldman and Goodrich, 2001). Freeman and Watts performed hundreds of procedures, and described relative success in the treatment of depression, agitation and aggression, although they cautioned its use should be limited to those patients with severe and refractory symptoms (Freeman and Watts, 1937). Unfortunately, this led to indiscriminate use of the lobotomy, partially in response to the lack of conventional treatments for mental illness, and also as a potential measure of reducing asylum overcrowding (Heller et al., 2008).

Freeman became emboldened by his initial success, and co-opted a transorbital technique developed by the Italian psychiatrist Amarro Fiamberti. This involved the insertion an instrument resembling an ice-pick, the orbitoclast, through the orbit into frontal lobe white matter, which allowed for lesioning via a sweeping motion in the coronal plane (Pressman, 1988). This technique was much quicker than the standard lobotomy and only required electroshock therapy for sedation; as a result, Freeman did not require an anesthetist, surgeon, or even proper sterile technique. In response, Watts and the neurosurgical community distanced themselves from this practice, and Freeman went on to perform thousands of procedures across America (Heller et al., 2008). The transorbital lobotomy was widely and eagerly adopted throughout America and parts of Europe, with tens of thousands of procedures performed across nearly two decades (Braslow, 1999). To illustrate the sheer popularity within the scientific community for psychosurgery at the time, the 1949 Nobel Prize in medicine or physiology was awarded in-part to Moniz for the leucotomy (Tierney, 2000).

### Stereotactic Neurosurgery and the Advent of “Minimalism”

Eventually, both professional and public opinion turned against the lobotomy as the associated morbidity and mortality became more apparent (Heller et al., 2008). A number of patients also developed a “post-leucotomy syndrome,” characterized by apathy, emotional blunting, and disinhibition. For these reasons, the medical community sought more scientifically rigorous surgical approaches focused on hypothesis-driven targeting with less aggressive lesioning and resections. The neurosurgeon William Scoville was an early proponent of these principles and performed selective undercutting of the orbitofrontal cortex (Scoville, 1949). Further improvements were realized with advancements in stereotactic neurosurgery, which superseded open and closed lesioning techniques.

The earliest stereotactic apparatus had previously been developed by Sir Victor Horsley and Robert Clark for animal experimentation (Clarke and Horsley, 2007), and its clinical translation was described by Ernest Spiegel and Henry Wycis (Gildenberg, 2001). Using x-ray ventriculography, the dorsomedial nucleus of the thalamus was targeted to treat agitation and psychosis (Spiegel et al., 1947). Further advances in psychosurgical stereotaxis led to the development of the stereotactic cingulotomy, capsulotomy, subcaudate tractotomy, and limbic leucotomy; rather than removing the entire frontal lobe to produce symptom relief, targeted lesions of white matter pathways and gray matter structures were found to be comparable in effect with fewer side effects (Lapidus et al., 2013). These procedures continue to be used for refractory psychiatric illness in contemporary practice. Additional targets, including the amygdala and hypothalamus, have also been described but are less commonly used.

The cingulotomy has been used for a variety of disorders, and involves lesioning of the cingulate gyrus and adjacent white matter fibers of cingulum bundle. In 1952, Sir Hugh Cairns described this technique for symptoms of anxiety and obsession (Whitty et al., 1952). The cingulotomy has since been used in the treatment of obsessive-compulsive disorder (OCD), treatment-resistant depression (TRD), and was similarly found to be effective for individuals suffering from chronic, intractable pain (Ballantine et al., 1967). The cingulotomy remains the most commonly performed psychosurgical procedure in North America (Lapidus et al., 2013), and its therapeutic effects are in-part believed to involve disruption of cortico-striato-thalamo-cortical circuitry.

Anterior capsulotomy has also been found to be effective for OCD, likely due to the disruption of frontothalamic fiber connections. It can be performed with radiofrequency coagulation, as originally described by Jean Talairach, or via gamma knife radiosurgery as described by Lars Leksell (Laitinen, 2001). Subcaudate tractotomy disrupts the orbitofrontal cortex from its thalamic and limbic connections, and its early use can be traced to Scoville’s orbitofrontal corticectomy (Scoville, 1949). Its adaptation into a stereotactic procedure can be attributed to Geoffrey Knight (Knight, 1965), and was initially performed via bilateral insertion of yttrium rods to cause radiation necrosis before transitioning to radiofrequency coagulation (Lapidus et al., 2013). Subcaudate tractotomy has been described for TRD and OCD, although cingulotomy and capsulotomy have largely surpassed its use in contemporary medicine (Lapidus et al., 2013). Lastly, the limbic leucotomy combines cingulotomy with tractotomy to target both frontothalamic and cingulate circuitry. First described by Desmond Kelly and Alan Richardson in 1973 (Kelly et al., 1973), it is likewise used for TRD and OCD. Ultimately, these techniques share a common theme of wide-spread neural network disruption.

## The Fall of Psychosurgery and the Rise of Pharmacology

The decline of psychosurgery in the 1950s can be attributed to numerous factors. The initial enthusiasm for frontal lobotomies was driven by widespread social acceptance and Freeman’s deft hand for advocacy and marketing. However, this also resulted in a laissez-faire attitude toward psychosurgery, with procedures being performed by non-neurosurgeons in inappropriate settings. This triggered professional criticism regarding the significant and largely underreported adverse events (Hoffman, 1949), and the lack of objectivity and scientific rigor. Furthermore, the public became aware of the undesirable consequences of lobotomies. Social attitudes were shaped by negative depictions in literature and film, including notable examples such as *One Flew Over the Cuckoo’s Nest*. It also became apparent that some institutionalized or incapacitated patients were lobotomized without informed consent, and procedures may have been performed on prisoners to address dysfunctional behavior as opposed to mental illness (Feldman and Goodrich, 2001). The controversy surrounding psychosurgery was instrumental in developing modern standards for research and ethics, with the passing of the National Research Act of 1974 and subsequent publication of the Belmont Report, which outlined the principles of informed consent and guidelines regarding the performance of medical procedures and research.

However, it was the evolution of pharmacotherapy that finally turned the tide against psychosurgery, particularly with the advent of lithium (Cade, 1949) and chlorpromazine (Lopez-Munoz et al., 2005) therapy. In particular, the use of chlorpromazine in the early 1950s provided an effective non-surgical treatment for severe aggression and psychosis. As a result, psychosurgery fell out of favor and psychopharmacology thrived as brisk sales prompted investment and further innovation. The development and approval for other antipsychotic and antidepressant agents soon followed, with evidence that medical therapy was more effective, safer, and cheaper than psychosurgery.

### Modern Psychopharmacological Treatments and Limitations to Medications

The standard first-line treatments for psychiatric disorders include medications and psychotherapy. Commonly used pharmacological agents are outlined in Table 1 (Gitlin, 2006; Marazziti and Consoli, 2010; Singer, 2010; Gao et al., 2015; Qaseem et al., 2016). Interestingly, there is significant overlap among psychiatric disorders and the pharmacological agents used in treatment, suggesting that these disorders likely involve widespread and shared circuitry and/or neurotransmitters.

**Table 1:** Summary of modern psychopharmacological agents.

Condition	Class	Medications	Indication	Symptoms targeted	Contraindications and/or Precautions^∗^	Side effects
Major Depressive Disorder	SSRI	Citalopram Escitalopram Fluoxetine Paroxetine Sertraline	First line	Attention, anxiety, depression, energy	Prolonged QT interval (primarily citalopram and escitalopram)	Anxiety, increased bleeding, diarrhea, dry mouth, headache, hyponatremia, insomnia, orthostasis, QTc prolongation, sedation, sexual dysfunction, weight gain
	SNRI	Desvenlafaxine Duloxetine Venlafaxine Levomilnacipran	First line	Attention, anxiety, depression, energy	Hepatic or renal impairment, narrow angle glaucoma	Headache, hypertension, insomnia, orthostasis, nausea, vomiting
	NDRI	Bupropion	First line	Appetite, depression, energy	Anorexia, alcohol or sedative withdrawal, Bulimia, Epilepsy	Anxiety, dry mouth, insomnia, seizures, weight loss
	NaSSA	Mirtazapine	First line	Appetite, anxiety, depression, sleep		Constipation, sedation, weight gain, aplastic anemia (rare)
	SM	Nefazodone Trazodone	Second line	Anxiety, depression, sleep	Hepatic impairment	Liver toxicity, priapism, orthostasis, sedation
	TCA	Amitriptyline Imipramine Nortriptyline Desipramine Doxepin Trimipramine	Second line	Anxiety, depression, energy, sedation	Acute recovery from myocardial infarction	Cardiac conduction effects, diaphoresis, dizziness, orthostasis, sedation, seizures, tremor, weight gain
	MAOI	Phenelzine Tranylcypromine	Thirdor fourth line	Anxiety, depression, energy	Congestive heart failure, pheochromocytoma, hepatic or renal impairment	Constipation, dry mouth, hypertensive crisis, insomnia, orthostasis, sedation, sexual dysfunction, weight gain
Bipolar Disorder	ATA	Aripiprazole Lurasidone Olanzapine Quetiapine Risperidone Ziprasidone Asenapine Clozapine Paliperidone	Acute or maintenance phase	Depression, mania, mood stabilization	Prolonged QT interval, recent myocardial infarction, heart failure, Parkinson disease	Agranulocytosis, akathisia, cataracts, cardiomyopathy, constipation, dystonia, hyperlipidemia, hyperprolactinemia, orthostasis, parkinsonism, sedation, seizures, sexual dysfunction, tardive dyskinesia, weight gain
	SSRI	Fluoxetine	Acute phase if combined with Olanzapine	Depression	(*see above*)	(*see above*)
	Metal	Lithium	Acute or maintenance phase	Depression, mania, mood stabilization, suicidality	Severe cardiac or renal impairment, severe dehydration/low sodium	Alopecia, confusion, diabetes insipidus, diarrhea, fatigue, hypocalcemia, hypothyroidism, nausea, t-wave changes, tremor, weakness, weight gain
	AC	Valproate, Carbamazepine Lamotrigine	Acute or maintenance phase	Depression, mania, mood stabilization	AV heart block, blood dyscrasia, hepatic impairment, hepatic porphyria	Alopecia, anemia, anorexia, diarrhea, hepatotoxicity, hyponatremia, nausea, sedation, Stevens-Johnson syndrome, thrombocytopenia, tremor, vomiting, weight gain
	Other	Thyroid hormone	Third line or augmentation	Depression		Tremor, headache, nausea, weight loss
	DA	Pramipexole	Acute phase	Depression	Severe cardiac or renal impairment	Dyskinesia, orthostasis, nausea
	DRI	Modafinil Armodafinil	Acute phase	Depression	Severe anxiety	Decreased appetite, headache
Obsessive Compulsive Disorder	SSRI	Fluoxetine Fluvoxamine Paroxetine Sertraline	First line	Obsessions, compulsions	(*see above*)	(*see above*)
	TCA	Clomipramine	Second line	Obsessions, compulsions	(*see above*)	(*see above*)
	ATA	Olanzapine Risperidone	Second line if combined with SSRI	Obsessions, compulsions	(*see above*)	(*see above*)
	TA	Haloperidol	Second line if combined with SSRI	Obsessions, compulsions	Long QT interval, narrow angle glaucoma, Parkinson disease	Akathisia, dystonia, parkinsonism, sedation, tardive dyskinesia, sexual dysfunction
Tourette Syndrome	A2A	Clonidine Guanfacine	First line	Tics, hyperactivity	Cardiac insufficiency	Dizziness, dry mouth, headache, irritability, orthostasis, sedation
	AC	Topiramate	First line	Tics	Recent alcohol use	Ataxia, confusion, diplopia, fatigue, sedation, weight loss
	TA	Haloperidol Pimozide	Second line	Tics	(*see above*)	Akathisia, constipation, dystonia, parkinsonism, sedation, tardive dyskinesia, sexual dysfunction, xerostomia
	ATA	Aripiprazole	Second line	Tics	(*see above*)	(*see above*)
	MAUI	Tetrabenazine	Third line	Tics	Depression, parkinsonism, suicidality	Anxiety, depression, hypotension, insomnia, nausea, sedation


*^∗^specific drug interactions not listed. SSRI, selective serotonin reuptake inhibitor; SNRI, serotonin norepinephrine reuptake inhibitor; NDRI, norepinephrine dopamine reuptake inhibitor; NaSSA, noradrenergic and specific serotonergic antidepressant; SM, serotonin modulator; TCA, tricyclic antidepressant; MAOI, monoamine oxidase inhibitor; ATA, atypical antipsychotic; AC, anticonvulsant; DA, dopamine agoinst; DRI, dopamine reuptake inhibitor; TA, typical antipsychotic; A2A, alpha II adrenergic agonist; MAUI, monoamine uptake inhibitor.*

However, treatment resistance or failure is common, necessitating alternative strategies. The first strategy is commonly to optimize medication dosing or scheduling, or switching to another therapeutic class. TRD, for example, refers to an inadequate response to at least one antidepressant trial, and can occur in up to half of patients (Fava, 2003). Even with optimized multi-modal therapy, up to 30% of patients with major depression are non-responders (Rush et al., 2006). Similarly, a significant proportion of patients with OCD (Greenberg et al., 2003), bipolar disorder (Geddes and Miklowitz, 2013), and malignant Tourette syndrome (Cheung et al., 2007) fail to respond to standard therapy, again suggesting that the underlying pathophysiology is likely complex and involves multiple networks. These treatment-resistant patients often have more severe symptoms, and higher rates of morbidity and mortality. As such, new therapeutic strategies are desperately required.

## Other Neuromodulation Therapies

Like psychosurgery, electroconvulsive therapy (ECT) was introduced before the advent of psychopharmacology. Despite its documented efficacy in the treatment of psychiatric disorders, its side effects on cognition and memory and misuse/abuse by some practitioners dramatically decreased its use before the 1980s. The realization of the imperfect effects of psychotropics “resuscitated” the use of ECT, although it is still considered under-utilized, primarily due to misconceptions regarding treatment (Bewernick and Schlaepfer, 2015). Although the exact mechanism is not understood, the induction of a generalized seizure and subsequent post-ictal suppression is effective for TRD (Sackeim et al., 2001) and bipolar disorder (Perugi et al., 2017), and may also be beneficial for OCD (Fontenelle et al., 2015).

Additional non-invasive therapies include magnetic seizure therapy, which focally induces the superficial cortex to produce seizures, repetitive transcranial magnetic stimulation, involving the pulse application of magnetic stimuli to alter cortical excitability, and vagal nerve stimulation, which sends electrical impulses to various brain regions via the solitary nucleus (Bewernick and Schlaepfer, 2015). The role for these alternative treatments remains unclear, as ECT is generally considered clinically superior (Dierckx et al., 2012; Berlim et al., 2013). However, the remission rates for TRD with ECT are reportedly from 35 to 53% (Schoeyen et al., 2015). Clearly, a significant number of patients will not respond to pharmacological and non-invasive neuromodulation treatments, suggesting that more invasive surgical interventions like deep brain stimulation (DBS) can be considered as a viable option for patients with psychiatric disorders who fail “all” available treatments.

## Necessity of Neuromodulation

With the advent of DBS, a reversible and adaptable technology, the use of lesioning procedures has lessened to some degree. Moreover, DBS has the potential to modulate numerous widespread networks. The most effective application of DBS has historically been in the treatment of movement disorders, however, its use has been trialed in a number of psychiatric conditions including Tourette syndrome (Vandewalle et al., 1999; Houeto et al., 2005; Kuhn et al., 2007; Maciunas et al., 2007; Neuner et al., 2009; Ackermans et al., 2011; Okun et al., 2013; Kefalopoulou et al., 2015), OCD (Nuttin et al., 1999; Mallet et al., 2008; Jimenez-Ponce et al., 2009; Denys et al., 2010; Greenberg et al., 2010; Huff et al., 2010), and depression (Mayberg et al., 2005; Malone et al., 2009; Kennedy et al., 2011; Bewernick et al., 2012; Holtzheimer et al., 2012; Lozano et al., 2012; Schlaepfer et al., 2013; Puigdemont et al., 2015; Bergfeld et al., 2016). Yet, OCD is the only psychiatric disorder for which DBS is currently FDA-approved under a humanitarian device exemption. A summary of relevant clinical trials, including their outcomes and adverse events is listed in Table 2.

**Table 2:** Deep brain stimulation for psychiatric disorders: selected references.

Psychiatric Disorder	DBS target	Authors and Year	Number of Patients	Outcomes	Therapy-Related adverse events^∗^
Tourette Syndrome	Thalamus	Vandewalle et al., 1999	1	Complete abolition of tics	None reported
		Maciunas et al., 2007	5	44% mean reduction in YGTSS	Acute psychotic episode
		Ackermans et al., 2011	6	49% mean reduction in YGTSS	Gaze disturbances, reduced energy
		Okun et al., 2013	5	Improvements in YGTSS, but did not meet main outcome measure of 50% improvement	None reported
	GPi	Houeto et al., 2005	1	65% mean reduction in YGTSS	Weight loss (attributed to neuroleptic withdrawal)
		Kefalopoulou et al., 2015	15	15.3% mean reduction in YGTSS	Hypomanic episode
	NAcc	Kuhn et al., 2007	1	41% mean reduction in YGTSS (NAcc+VC/VS)	None reported
		Neuner et al., 2009	1	86% mean reduction in YGTSS	None reported
Obsessive Compulsive Disorder	VC/VS	Nuttin et al., 1999	4	Beneficial effects in 75% of patients	None reported
		Greenberg et al., 2010	26	≥ 25% reduction in YBOCS in 73% of patients	Hypomanic episode, irritability, suicidal ideation
	NAcc	Denys et al., 2010	16	25% mean reduction in YBOCS compared to sham	Forgetfulness, word finding difficulties
		Huff et al., 2010	10	≥ 25% mean reduction in YBOCS in 50% of patients	Dysesthesia, headache, hypomanic episode, suicidal ideations
	STN	Mallet et al., 2008	8	32% mean reduction in YBOCS compared to sham	Ambulatory difficulty, hypomanic episode, dyskinesia, dysphagia
	ITP	Jimenez-Ponce et al., 2009	5	51% mean reduction in YBOCS	Confusion, dysautonomia
Depression	NAcc	Bewernick et al., 2012	11	45% sustained response after 4 years	Suicide (non-responder)
	SCC	Kennedy et al., 2011	20	64% response rate after 3–6 years	Suicide
		Puigdemont et al., 2015	5	Reduction of HDRS during active stimulation compared to sham in 80% of patients	None reported
		Lozano et al., 2012	21	29% reduction in HDRS	Nausea, vomiting, suicide
		Holtzheimer et al., 2012	17	92% response and 58% remission after 2 years	Two suicide attempts (stress association)
		Riva-Posse et al., 2018	11	Preoperative deterministic tractography matched with postoperative probabilistic tractography; 82% response and 55% remission after 1 year	None reported
	VC/VS	Malone et al., 2009	15	53% response and 40% remission at last follow-up	Hypomanic episode, syncope, disinhibition
		Bergfeld et al., 2016	25	40% response, 24% partial response and 20% remission after optimization phase	Suicidal ideation and attempts, nocturia, blurred vision, disinhibition
	MFB	Schlaepfer et al., 2013	7	86% response and 57% remission after 12–33 weeks	Dizziness, oculomotor disturbances, increasing sweating


*^∗^not including device or implantation-related complications. GPi, globus pallidus internus; HDRS, Hamilton Rating Scale for Depression; ITP, Inferior thalamic peduncle; MFB, medial forebrain bundle; NAcc, nucleus accumbens; SCC, subcallosal cingulate cortex; STN, subthalamic nucleus; VC/VS, anterior limb of the internal capsule/ventral capsule/ventral striatum; YBOCS, Yale-Brown Obsessive Compulsive Scale; YGTSS, Yale Global Tic Severity Scale.*

Although DBS has shown efficacy in patients with treatment-resistant major depression and bipolar depression (Holtzheimer et al., 2012), the investigation of DBS for such diseases at TRD in larger scale randomized trials has been less successful (Dougherty et al., 2015; Holtzheimer et al., 2017). This may in part be due to the unclear determination of targets that would effectively produce the same results seen with lesioning techniques. Traditionally, DBS targets have been selected based on neuroimaging findings and historical results of lesioning procedures, though clearly further hypothesis-directed targeting studies are needed.

## Future Directions

There are numerous limitations to the current understanding and treatment of psychiatric disorders. These are complex disease states which cannot be localized to distinct anatomical correlates, and are influenced by cognitive, behavioral and emotional factors. As such, the contemporary understanding of psychiatric disease may be attributable to a dysfunction of networks rather than an isolated structural or neurotransmitter abnormality. This interpretation can be traced to seminal works including the description of the Papez and cortico-striato-thalamocortical circuits, which postulate that functional subdivisions influence various facets of behavior (Alexander et al., 1986; Papez, 1995).

If these diseases are truly abnormalities in wide-spread white matter connections and brain circuitry, then a better understanding of such pathways in the human brain is essential. While data from animal studies remains the gold-standard for the evaluation of white matter fiber tracts, these models may not accurately translate to the human correlate with such complex behavioral and cognitive processing. Moreover, although improvements in neuroimaging have been fundamental in advancing our understanding of the underlying neurobiology and circuitry involved in psychiatric disorders, these also have limitations in their ability to precisely and accurately allow the visualization of these connections. Innovations in neuro-imaging techniques will be necessary to help identify radiographic biomarkers indicative of a particular disease entity. Methodological innovations that may improve the accuracy of imaging technologies include advances in machine learning, optimization of signal prediction error, and the development of novel imaging algorithms (Maier-Hein et al., 2017). Furthermore, there is likely significant patient variability regarding this underlying circuitry. Riva-Posse et al. have previously demonstrated that patient responders to DBS for TRD shared similar fiber pathways that were being stimulated (Riva-Posse et al., 2014). Their subsequent prospective study utilizing probabilistic tractography resulted in a high patient response rate, suggesting an important role for targeting based on patient-specific data (Riva-Posse et al., 2018).

Advances in DBS technology and transition to a “closed loop” system may hold promise for the treatment of psychiatric disorders based on patient or disease-specific biomarkers. Conventional “open loop” stimulation requires the physician to adjust stimulation parameters based on subjective evaluations, whereas a closed-loop system adjusts stimulation parameters based on direct measurement of a neural biomarker (Lo and Widge, 2017). In Parkinson’s disease, pathological beta band activity may represent an essential biomarker for adaptive stimulation (Moraud et al., 2018). However, biomarker identification in psychiatric disease states will prove to be more challenging due to clinical heterogeneity, and may be dependent on identifying commonalities among disease phenotypes (Bilge et al., 2018). The implantation of DBS electrodes allows for both acute (microelectrode) and chronic (macroelectrode) recordings to identify potential electrophysiological biomarkers in psychiatric disease states. Such recordings may provide insight into the electrophysiological correlates of acute symptomatology, and also long-term changes as a result of the disease process or chronic DBS therapy.

There is emerging evidence that local field potential activity from various brain regions may have distinct patterns in psychiatric disorders (Neumann et al., 2014; Merkl et al., 2016), and that invasive electrophysiology may allow for circuitry identification and modulation in selected patients (Widge et al., 2017). In a pre-clinical study of mice prone to binge-eating, Wu et al. observed that closed-loop stimulation of the nucleus accumbens disrupted rewarding behavior (Wu et al., 2018). Specifically, the identified biomarker was pathological delta band activity from local field potential recordings. Although promising, closed-loop stimulation has yet to be clinically validated in psychiatric disorders.

It has also been suggested that the lack of treatment success of DBS for psychiatric disorders in larger clinical studies may be attributable to the clinical trial design itself (Dougherty, 2018). Whereas the efficacy of DBS for movement disorders can be reliably measured based on objective markers, the diagnostic criteria and interpretation of psychiatric disorders can be more variable and complex, and the clinical response may take longer to manifest. As such, clinical trial design should take into consideration different therapeutic endpoints and/or a longer trial period.

Ultimately, the contemporary use of neuromodulation for mental illness will need to focus on a hypothesis-driven approach to target selection and will likely incorporate multiple targeting methods using novel technologies to produce effective results (Riva-Posse et al., 2014, 2018). Patient heterogeneity should not be under-valued, and an individualized approach may also be required, be it symptom-directed, patient-directed, or both (Holtzheimer and Mayberg, 2011).

## Conclusion

The modern resurgence of surgical therapies for psychiatric disorders must respect the lessons learned from the early and mid-twentieth century, particularly the ethical and social ramifications of poor scientific rigor. The treatment of psychiatric patients continues to be challenging, especially in those patients deemed incapable of making informed decisions. For these reasons, the practice of psychosurgery should embrace a multidisciplinary approach to diagnosis and management. The future of psychiatric treatments will undoubtedly involve concurrent developments in the fields of behavioral neuroscience, neuroimaging, psychopharmacology, and neurosurgery.

## Author Contributions

MS and JS participated in manuscript conceptualization, design, and preparation. MS, EH, KG, JM, and JS provided critical revisions of the manuscript and reviewed the submitted version. KG, JM, and JS provided administrative support.

## Conflict of Interest Statement

JS receives grant support from the National Institutes of Health (2KL2TR000440; clinicaltrials.gov, NCT02655978) and is on the Scientific Advisory Board for Koh Young Inc. KG was on a speakers bureau of AstraZeneca, Pfizer and Sunovion, and an Advisory Board of Sunovion and Otsuka, and received grant supports from AstraZeneca, Brain and Behavior Research Foundation, and Cleveland Foundation. The remaining authors declare that the research was conducted in the absence of any commercial or financial relationships that could be construed as a potential conflict of interest.

## References

[B1] Ackermans L. Duits A. van der Linden C. Tijssen M. Schruers K. Temel Y. (2011). Double-blind clinical trial of thalamic stimulation in patients with tourette syndrome. *Brain* 134(Pt 3), 832–844. 10.1093/brain/awq380 21354977

[B2] Alexander G. E. DeLong M. R. Strick P. L. (1986). Parallel organization of functionally segregated circuits linking basal ganglia and cortex. *Annu. Rev. Neurosci.* 9 357–381. 10.1146/annurev.ne.09.030186.002041 3085570

[B3] Alt K. W. Jeunesse C. Buitrago-Tellez C. H. Wachter R. Boes E. Pichler S. L. (1997). Evidence for stone age cranial surgery. *Nature* 387:360. 10.1038/387360a0 9163419

[B4] Ballantine H. T. Jr. Cassidy W. L. Flanagan N. B. (1967). Stereotaxic anterior cingulotomy for neuropsychiatric illness and intractable pain. *J. Neurosurg.* 26 488–495. 10.3171/jns.1967.26.5.0488 5337782

[B5] Bergfeld I. O. Mantione M. Hoogendoorn M. L. Ruhe H. G. Notten P. van Laarhoven J. (2016). Deep brain stimulation of the ventral anterior limb of the internal capsule for treatment-resistant depression: a randomized clinical trial. *JAMA Psychiatry* 73 456–464. 10.1001/jamapsychiatry.2016.0152 27049915

[B6] Berlim M. T. Van den Eynde F. Daskalakis Z. J. (2013). Efficacy and acceptability of high frequency repetitive transcranial magnetic stimulation (rTMS) versus electroconvulsive therapy (ECT) for major depression: a systematic review and meta-analysis of randomized trials. *Depress. Anxiety* 30 614–623. 10.1002/da.22060 23349112

[B7] Bewernick B. Schlaepfer T. E. (2015). Update on neuromodulation for treatment-resistant depression. *F1000Res* 4:F1000 Faculty Rev-1389. 10.12688/f1000research.6633.1 26918135 PMC4754006

[B8] Bewernick B. H. Kayser S. Sturm V. Schlaepfer T. E. (2012). Long-term effects of nucleus accumbens deep brain stimulation in treatment-resistant depression: evidence for sustained efficacy. *Neuropsychopharmacology* 37 1975–1985. 10.1038/npp.2012.44 22473055 PMC3398749

[B9] Bilge M. T. Gosai A. K. Widge A. S. (2018). Deep brain stimulation in psychiatry: mechanisms, models, and next-generation therapies. *Psychiatr. Clin. North Am.* 41 373–383. 10.1016/j.psc.2018.04.003 30098651 PMC6092041

[B10] Braslow J. T. (1999). History and evidence-based medicine: lessons from the history of somatic treatments from the 1900s to the 1950s. *Ment. Health Serv. Res.* 1 231–240. 10.1023/A:1022325508430 11256729

[B11] Cade J. F. (1949). Lithium salts in the treatment of psychotic excitement. *Med. J. Aust.* 2 349–352. 18142718 10.1080/j.1440-1614.1999.06241.x

[B12] Cheung M. Y. Shahed J. Jankovic J. (2007). Malignant tourette syndrome. *Mov. Disord.* 22 1743–1750. 10.1002/mds.21599 17566119

[B13] Clarke R. H. Horsley V. (2007). THE CLASSIC: on a method of investigating the deep ganglia and tracts of the central nervous system (cerebellum). Br Med J 1906:1799-1800. *Clin. Orthop. Relat. Res.* 463 3–6. 10.1097/BLO.0b013e31814d4d99 17912057

[B14] Damasio H. Grabowski T. Frank R. Galaburda A. M. Damasio A. R. (1994). The return of phineas gage: clues about the brain from the skull of a famous patient. *Science* 264 1102–1105. 10.1126/science.8178168 8178168

[B15] Denys D. Mantione M. Figee M. van den Munckhof P. Koerselman F. Westenberg H. (2010). Deep brain stimulation of the nucleus accumbens for treatment-refractory obsessive-compulsive disorder. *Arch. Gen. Psychiatry* 67 1061–1068. 10.1001/archgenpsychiatry.2010.122 20921122

[B16] Dierckx B. Heijnen W. T. van den Broek W. W. Birkenhager T. K. (2012). Efficacy of electroconvulsive therapy in bipolar versus unipolar major depression: a meta-analysis. *Bipolar Disord.* 14 146–150. 10.1111/j.1399-5618.2012.00997.x 22420590

[B17] Dimopoulos V. G. Robinson J. S. 3rd Fountas K. N. (2008). The pearls and pitfalls of skull trephination as described in the hippocratic treatise “On Head Wounds”. *J. Hist. Neurosci.* 17 131–140. 10.1080/09647040701296770 18421631

[B18] Dougherty D. D. (2018). Deep brain stimulation: clinical applications. *Psychiatr. Clin. North Am.* 41 385–394. 10.1016/j.psc.2018.04.004 30098652

[B19] Dougherty D. D. Rezai A. R. Carpenter L. L. Howland R. H. Bhati M. T. O’Reardon J. P. (2015). A randomized sham-controlled trial of deep brain stimulation of the ventral capsule/ventral striatum for chronic treatment-resistant depression. *Biol. Psychiatry* 78 240–248. 10.1016/j.biopsych.2014.11.023 25726497

[B20] Fava M. (2003). Diagnosis and definition of treatment-resistant depression. *Biol. Psychiatry* 53 649–659. 10.1016/S0006-3223(03)00231-2 12706951

[B21] Feldman R. P. Goodrich J. T. (2001). Psychosurgery: a historical overview. *Neurosurgery* 48 647–657; discussion 657–649. 10.1097/00006123-200103000-00041 11270556

[B22] Fontenelle L. F. Coutinho E. S. Lins-Martins N. M. Fitzgerald P. B. Fujiwara H. Yucel M. (2015). Electroconvulsive therapy for obsessive-compulsive disorder: a systematic review. *J. Clin. Psychiatry* 76 949–957. 10.4088/JCP.14r09129 25844888

[B23] Freeman W. Watts J. W. (1937). Prefrontal lobotomy in the treatment of mental disorders. *South Med. J.* 30 23–31. 10.1097/00007611-193701000-00005

[B24] Fulton J. F. Jacobson C. F. (1935). The functions of the frontal lobes: a comparative study in monkeys, chimpanzees and man. *Adv. Mod. Biol.* 4 113–123. 16782503

[B25] Gao K. Kemp D.E. Wu R. Calabrese J.R. (2015). “Mood stabilizers,” in *Psychiatry*, eds Tasman A. Kay J. Lieberman A. First M.B. Riba M.B. (Chichester: John Wiley & Sons, Ltd).

[B26] Geddes J. R. Miklowitz D. J. (2013). Treatment of bipolar disorder. *Lancet* 381 1672–1682. 10.1016/S0140-6736(13)60857-0 23663953 PMC3876031

[B27] Gildenberg P. L. (2001). Spiegel and wycis the early years. *Stereotact. Funct. Neurosurg.* 77 11–16. 10.1159/000064587 12378049

[B28] Gitlin M. (2006). Treatment-resistant bipolar disorder. *Mol. Psychiatry* 11 227–240. 10.1038/sj.mp.4001793 16432528

[B29] Greenberg B. D. Gabriels L. A. Malone D. A. Jr. Rezai A. R. Friehs G. M. (2010). Deep brain stimulation of the ventral internal capsule/ventral striatum for obsessive-compulsive disorder: worldwide experience. *Mol. Psychiatry* 15 64–79. 10.1038/mp.2008.55 18490925 PMC3790898

[B30] Greenberg B. D. Price L. H. Rauch S. L. Friehs G. Noren G. Malone D. (2003). Neurosurgery for intractable obsessive-compulsive disorder and depression: critical issues. *Neurosurg. Clin. N. Am.* 14 199–212. 10.1016/S1042-3680(03)00005-6 12856488

[B31] Heller A. C. Amar A. P. Liu C. Y. Apuzzo M. L. (2008). Surgery of the mind and mood: a mosaic of issues in time and evolution. *Neurosurgery* 62(6 Suppl. 3), 921–940. 10.1227/01.neu.0000333761.30877.8d 18695579

[B32] Hoffman J. L. (1949). Clinical observations concerning schizophrenic patients treated by prefrontal leukotomy. *N. Engl. J. Med.* 241 233–236. 10.1056/NEJM194908112410604 18138676

[B33] Holtzheimer P. E. Husain M. M. Lisanby S. H. Taylor S. F. Whitworth L. A. McClintock S. (2017). Subcallosal cingulate deep brain stimulation for treatment-resistant depression: a multisite, randomised, sham-controlled trial. *Lancet Psychiatry* 4 839–849. 10.1016/S2215-0366(17)30371-1 28988904

[B34] Holtzheimer P. E. Kelley M. E. Gross R. E. Filkowski M. M. Garlow S. J. Barrocas A. (2012). Subcallosal cingulate deep brain stimulation for treatment-resistant unipolar and bipolar depression. *Arch. Gen. Psychiatry* 69 150–158. 10.1001/archgenpsychiatry.2011.1456 22213770 PMC4423545

[B35] Holtzheimer P. E. Mayberg H. S. (2011). Stuck in a rut: rethinking depression and its treatment. *Trends Neurosci.* 34 1–9. 10.1016/j.tins.2010.10.004 21067824 PMC3014414

[B36] Houeto J. L. Karachi C. Mallet L. Pillon B. Yelnik J. Mesnage V. (2005). Tourette’s syndrome and deep brain stimulation. *J. Neurol. Neurosurg. Psychiatry* 76 992–995. 10.1136/jnnp.2004.043273 15965209 PMC1739716

[B37] Huff W. Lenartz D. Schormann M. Lee S. H. Kuhn J. Koulousakis A. (2010). Unilateral deep brain stimulation of the nucleus accumbens in patients with treatment-resistant obsessive-compulsive disorder: outcomes after one year. *Clin. Neurol. Neurosurg* 112 137–143. 10.1016/j.clineuro.2009.11.006 20006424

[B38] Jimenez-Ponce F. Velasco-Campos F. Castro-Farfan G. Nicolini H. Velasco A. L. Salin-Pascual R. (2009). Preliminary study in patients with obsessive-compulsive disorder treated with electrical stimulation in the inferior thalamic peduncle. *Neurosurgery* 65(6 Suppl), 203–209; discussion 209. 10.1227/01.NEU.0000345938.39199.90 19934996

[B39] Kefalopoulou Z. Zrinzo L. Jahanshahi M. Candelario J. Milabo C. Beigi M. (2015). Bilateral globus pallidus stimulation for severe Tourette’s syndrome: a double-blind, randomised crossover trial. *Lancet Neurol.* 14 595–605. 10.1016/S1474-4422(15)00008-3 25882029

[B40] Kelly D. Richardson A. Mitchell-Heggs N. (1973). Stereotactic limbic leucotomy: neurophysiological aspects and operative technique. *Br. J. Psychiatry* 123 133–140. 10.1192/bjp.123.2.133 4582234

[B41] Kennedy S. H. Giacobbe P. Rizvi S. J. Placenza F. M. Nishikawa Y. Mayberg H. S. (2011). Deep brain stimulation for treatment-resistant depression: follow-up after 3 to 6 years. *Am. J. Psychiatry* 168 502–510. 10.1176/appi.ajp.2010.10081187 21285143

[B42] Knight G. (1965). Stereotactic Tractotomy in the Surgical Treatment of Mental Illness. *J. Neurol. Neurosurg. Psychiatry* 28 304–310. 10.1136/jnnp.28.4.304 14338119 PMC495909

[B43] Kuhn J. Lenartz D. Mai J. K. Huff W. Lee S. H. Koulousakis A. (2007). Deep brain stimulation of the nucleus accumbens and the internal capsule in therapeutically refractory Tourette-syndrome. *J. Neurol.* 254 963–965. 10.1007/s00415-006-0404-8 17410328

[B44] Laitinen L. V. (2001). Psychosurgery. *Stereotact. Funct. Neurosurg.* 76 239–242. 10.1159/000066724 12378102

[B45] Lapidus K. A. Kopell B. H. Ben-Haim S. Rezai A. R. Goodman W. K. (2013). History of psychosurgery: a psychiatrist’s perspective. *World Neurosurg.* 80 S27 e1–16. 10.1016/j.wneu.2013.02.053 23419707

[B46] Lo M. C. Widge A. S. (2017). Closed-loop neuromodulation systems: next-generation treatments for psychiatric illness. *Int. Rev. Psychiatry* 29 191–204. 10.1080/09540261.2017.1282438 28523978 PMC5461950

[B47] Lopez-Munoz F. Alamo C. Cuenca E. Shen W. W. Clervoy P. Rubio G. (2005). History of the discovery and clinical introduction of chlorpromazine. *Ann. Clin. Psychiatry* 17 113–135. 10.1080/10401230591002002 16433053

[B48] Lozano A. M. Giacobbe P. Hamani C. Rizvi S. J. Kennedy S. H. Kolivakis T. T. (2012). A multicenter pilot study of subcallosal cingulate area deep brain stimulation for treatment-resistant depression. *J. Neurosurg.* 116 315–322. 10.3171/2011.10.JNS102122 22098195

[B49] Maciunas R. J. Maddux B. N. Riley D. E. Whitney C. M. Schoenberg M. R. Ogrocki P. J. (2007). Prospective randomized double-blind trial of bilateral thalamic deep brain stimulation in adults with Tourette syndrome. *J. Neurosurg.* 107 1004–1014. 10.3171/JNS-07/11/1004 17977274

[B50] Maier-Hein K. H. Neher P. F. Houde J. C. Cote M. A. Garyfallidis E. Zhong J. (2017). The challenge of mapping the human connectome based on diffusion tractography. *Nat. Commun.* 8:1349. 10.1038/s41467-017-01285-x 29116093 PMC5677006

[B51] Mallet L. Polosan M. Jaafari N. Baup N. Welter M. L. Fontaine D. (2008). Subthalamic nucleus stimulation in severe obsessive-compulsive disorder. *N. Engl. J. Med.* 359 2121–2134. 10.1056/NEJMoa0708514 19005196

[B52] Malone D. A. Jr. Dougherty D. D. Rezai A. R. Carpenter L. L. Friehs G. M. (2009). Deep brain stimulation of the ventral capsule/ventral striatum for treatment-resistant depression. *Biol. Psychiatry* 65 267–275. 10.1016/j.biopsych.2008.08.029 18842257 PMC3486635

[B53] Marazziti D. Consoli G. (2010). Treatment strategies for obsessive-compulsive disorder. *Expert Opin. Pharmacother.* 11 331–343. 10.1517/14656560903446948 20102301

[B54] Mayberg H. S. Lozano A. M. Voon V. McNeely H. E. Seminowicz D. Hamani C. (2005). Deep brain stimulation for treatment-resistant depression. *Neuron* 45 651–660. 10.1016/j.neuron.2005.02.014 15748841

[B55] Merkl A. Neumann W. J. Huebl J. Aust S. Horn A. Krauss J. K. (2016). Modulation of beta-band activity in the subgenual anterior cingulate cortex during emotional empathy in treatment-resistant depression. *Cereb. Cortex* 26 2626–2638. 10.1093/cercor/bhv100 25994959

[B56] Moniz E. (1994). Prefrontal leucotomy in the treatment of mental disorders. 1937. Am J Psychiatry 151(6 Suppl), 236–239. 10.1176/ajp.151.6.236 8192205

[B57] Moraud E. M. Tinkhauser G. Agrawal M. Brown P. Bogacz R. (2018). Predicting beta bursts from local field potentials to improve closed-loop DBS paradigms in Parkinson’s patients. *Conf. Proc. IEEE Eng. Med. Biol. Soc.* 2018 3766–3796. 10.1109/EMBC.2018.8513348 30441186 PMC6277014

[B58] Neumann W. J. Huebl J. Brucke C. Gabriels L. Bajbouj M. Merkl A. (2014). Different patterns of local field potentials from limbic DBS targets in patients with major depressive and obsessive compulsive disorder. *Mol. Psychiatry* 19 1186–1192. 10.1038/mp.2014.2 24514569 PMC4813757

[B59] Neuner I. Podoll K. Lenartz D. Sturm V. Schneider F. (2009). Deep brain stimulation in the nucleus accumbens for intractable tourette’s syndrome: follow-up report of 36 months. *Biol. Psychiatry* 65 e5–6. 10.1016/j.biopsych.2008.09.030 19006786

[B60] Nuttin B. Cosyns P. Demeulemeester H. Gybels J. Meyerson B. (1999). Electrical stimulation in anterior limbs of internal capsules in patients with obsessive-compulsive disorder. *Lancet* 354:1526 10.1016/S0140-6736(99)02376-4 10551504

[B61] Okun M. S. Foote K. D. Wu S. S. Ward H. E. Bowers D. Rodriguez R. L. (2013). A trial of scheduled deep brain stimulation for Tourette syndrome: moving away from continuous deep brain stimulation paradigms. *JAMA Neurol.* 70 85–94. 10.1001/jamaneurol.2013.580 23044532

[B62] Papez J. W. (1995). A proposed mechanism of emotion. 1937. *J. Neuropsychiatry Clin. Neurosci.* 7 103–112. 10.1176/jnp.7.1.103 7711480

[B63] Perugi G. Medda P. Toni C. Mariani M. G. Socci C. Mauri M. (2017). The role of electroconvulsive therapy (ect) in bipolar disorder: effectiveness in 522 patients with bipolar depression, mixed-state, mania and catatonic features. *Curr. Neuropharmacol.* 15 359–371. 10.2174/1570159X14666161017233642 28503107 PMC5405614

[B64] Pressman J. D. (1988). Richard H. Shryock medal essay. Sufficient promise: John F. Fulton and the origins of psychosurgery. *Bull. Hist. Med.* 62 1–22. 3285920

[B65] Puigdemont D. Portella M. Perez-Egea R. Molet J. Gironell A. de Diego-Adelino J. (2015). A randomized double-blind crossover trial of deep brain stimulation of the subcallosal cingulate gyrus in patients with treatment-resistant depression: a pilot study of relapse prevention. *J. Psychiatry Neurosci.* 40 224–231. 10.1503/jpn.130295 25652752 PMC4478055

[B66] Qaseem A. Barry M. J. Kansagara D. (2016). Nonpharmacologic versus pharmacologic treatment of adult patients with major depressive disorder: a clinical practice guideline from the american college of physicians. *Ann. Intern. Med.* 164 350–359. 10.7326/M15-2570 26857948

[B67] Riva-Posse P. Choi K. S. Holtzheimer P. E. Crowell A. L. Garlow S. J. Rajendra J. K. (2018). A connectomic approach for subcallosal cingulate deep brain stimulation surgery: prospective targeting in treatment-resistant depression. *Mol. Psychiatry* 23 843–849. 10.1038/mp.2017.59 28397839 PMC5636645

[B68] Riva-Posse P. Choi K. S. Holtzheimer P. E. McIntyre C. C. Gross R. E. Chaturvedi A. (2014). Defining critical white matter pathways mediating successful subcallosal cingulate deep brain stimulation for treatment-resistant depression. *Biol. Psychiatry* 76 963–969. 10.1016/j.biopsych.2014.03.029 24832866 PMC4487804

[B69] Robison R. A. Taghva A. Liu C. Y. Apuzzo M. L. (2013). Surgery of the mind, mood, and conscious state: an idea in evolution. *World Neurosurg.* 80 S2–26. 10.1016/j.wneu.2013.08.002 23916496

[B70] Rush A. J. Trivedi M. H. Wisniewski S. R. Nierenberg A. A. Stewart J. W. Warden D. (2006). Acute and longer-term outcomes in depressed outpatients requiring one or several treatment steps: a STAR^∗^D report. *Am. J. Psychiatry* 163 1905–1917. 10.1176/ajp.2006.163.11.1905 17074942

[B71] Sackeim H. A. Haskett R. F. Mulsant B. H. Thase M. E. Mann J. J. Pettinati H. M. (2001). Continuation pharmacotherapy in the prevention of relapse following electroconvulsive therapy: a randomized controlled trial. *JAMA* 285 1299–1307. 10.1001/jama.285.10.1299 11255384

[B72] Salcman M. (2006). The cure of folly or The operation for the stone by Hieronymus Bosch (C. 1450-1516). *Neurosurgery* 59 935–937. 10.1227/01.NEU.0000242109.97950.9F 17038958

[B73] Schlaepfer T. E. Bewernick B. H. Kayser S. Madler B. Coenen V. A. (2013). Rapid effects of deep brain stimulation for treatment-resistant major depression. *Biol. Psychiatry* 73 1204–1212. 10.1016/j.biopsych.2013.01.034 23562618

[B74] Schoeyen H. K. Kessler U. Andreassen O. A. Auestad B. H. Bergsholm P. Malt U. F. (2015). Treatment-resistant bipolar depression: a randomized controlled trial of electroconvulsive therapy versus algorithm-based pharmacological treatment. *Am. J. Psychiatry* 172 41–51. 10.1176/appi.ajp.2014.13111517 25219389

[B75] Scoville W. B. (1949). Selective cortical undercutting as a means of modifying and studying frontal lobe function in man. *J. Neurosurg.* 6 65–73. 10.3171/jns.1949.6.1.0065 18106833

[B76] Simpson D. (2005). Phrenology and the neurosciences: contributions of F. J. Gall and J. G. Spurzheim. *ANZ J. Surg.* 75 475–482. 10.1111/j.1445-2197.2005.03426.x 15943741

[B77] Singer H. S. (2010). Treatment of tics and tourette syndrome. *Curr. Treat. Options Neurol.* 12 539–561. 10.1007/s11940-010-0095-4 20848326

[B78] Spiegel E. A. Wycis H. T. Marks M. Lee A. J. (1947). Stereotaxic apparatus for operations on the human brain. *Science* 106 349–350. 10.1126/science.106.2754.349 17777432

[B79] Stone J. L. (2001). Dr. Gottlieb Burckhardt–the pioneer of psychosurgery. *J. Hist. Neurosci.* 10 79–92. 10.1076/jhin.10.1.79.5634 11446267

[B80] Stone J. L. Miles M. L. (1990). Skull trepanation among the early Indians of Canada and the United States. *Neurosurgery* 26 1015–1019; discussion 1019–1020. 10.1227/00006123-199006000-00016 2194137

[B81] Tierney A. J. (2000). Egas moniz and the origins of psychosurgery: a review commemorating the 50th anniversary of Moniz’s nobel prize. *J. Hist. Neurosci.* 9 22–36. 10.1076/0964-704X(200004)9:1;1-2;FT022 11232345

[B82] Vandewalle V. van der Linden C. Groenewegen H. J. Caemaert J. (1999). Stereotactic treatment of gilles de la tourette syndrome by high frequency stimulation of thalamus. *Lancet* 353:724. 10.1016/S0140-6736(98)05964-9 10073521

[B83] Whitty C. W. Duffield J. E. Tov P. M. Cairns H. (1952). Anterior cingulectomy in the treatment of mental disease. *Lancet* 1 475–481. 10.1016/S0140-6736(52)90051-2 14898782

[B84] Widge A. S. Ellard K. K. Paulk A. C. Basu I. Yousefi A. Zorowitz S. (2017). Treating refractory mental illness with closed-loop brain stimulation: progress towards a patient-specific transdiagnostic approach. *Exp. Neurol.* 287(Pt 4), 461–472. 10.1016/j.expneurol.2016.07.021 27485972

[B85] Wu H. Miller K. J. Blumenfeld Z. Williams N. R. Ravikumar V. K. Lee K. E. (2018). Closing the loop on impulsivity via nucleus accumbens delta-band activity in mice and man. *Proc. Natl. Acad. Sci. U.S.A.* 115 192–197. 10.1073/pnas.1712214114 29255043 PMC5776799

